# Advance directives in German memory clinics

**DOI:** 10.1007/s00391-025-02455-z

**Published:** 2025-06-27

**Authors:** Daniel Garmann, Christina Abele, Stefanie Baisch, Jonas Karneboge, Gregor Lindl, Janina Florack, Anna Theile-Schürholz, Julia Haberstroh

**Affiliations:** 1 Department of Psychology, Psychological Aging Research, University of Siegen, Adolf-Reichwein-Str. 2a, 57068 Siegen, Germany; 2 Klinikum Siegen, Siegen, Germany; 3 University Medical Center of the Johannes Gutenberg University Mainz, Mainz, Germany; 4 Department of Psychiatry, Psychosomatic and Psychotherapy, Goethe University Frankfurt, University Hospital, Frankfurt am Main, Germany

**Keywords:** Advance care planning, Patient autonomy, Dementia, Informed consent, End-of-life decisions, Gesundheitliche Vorausplanung, Patientenautonomie, Demenz, Informierte Einwilligung, Entscheidungen am Lebensende

## Abstract

People have the right to accept or refuse medical treatment provided they give voluntary, prior and informed consent. When capacity to consent is impaired, autonomy can be preserved through a clear, legally valid advance directive. Cognitive impairments, such as dementia, increase the risk of compromised capacity to consent. This study examines the prevalence of advance directives among patients at German memory clinics and explores predictors influencing their presence, focusing on compliance with informed consent standards.

Using an observational cross-sectional design, 289 outpatients were assessed. Data included the presence of advance directives, circumstances of their creation and the patient characteristics age, gender, education, cognitive status, physical and mental health, need for autonomy in medical decision-making and health literacy. Descriptive statistics were calculated, and multivariable logistic regression models were applied to analyze the data.

The results indicate that age is the strongest predictor for the presence of advance directives. Beyond age, specific events or experiences, rather than personal traits, may prompt individuals to create advance directives.

**Supplementary Information:**

The online version of this article (10.1007/s00391-025-02455-z) contains supplementary material, which is available to authorized users.

## Abstract (de)

Jede Person hat das Recht, medizinische Behandlungen anzunehmen oder abzulehnen, sofern sie freiwillig, informiert und im Voraus einwilligt. Bei eingeschränkter Einwilligungsfähigkeit kann Autonomie durch eine eindeutige und rechtlich gültige Patientenverfügung gewahrt werden. Kognitive Beeinträchtigungen, wie sie etwa bei Demenz auftreten, erhöhen das Risiko einer eingeschränkten Einwilligungsfähigkeit. Diese Studie untersucht die Prävalenz von Patientenverfügungen bei Patienten in deutschen Gedächtnisambulanzen sowie Prädiktoren für deren Vorhandensein unter Berücksichtigung des Informed-Consent-Standards.

In einer Querschnittsstudie mit 289 Patienten wurden das Vorhandensein von Patientenverfügungen, die Umstände ihrer Erstellung und patientenbezogene Merkmale wie Alter, Geschlecht, Bildung, kognitiver Status, körperliche und psychische Gesundheit, Autonomiebedürfnis und Gesundheitskompetenz erhoben. Deskriptive Statistiken und multivariable logistische Regressionsmodelle kamen zur Analyse zum Einsatz.

Die Ergebnisse zeigen, dass Alter der stärkste Prädiktor für das Vorliegen von Patientenverfügungen ist, und konkrete Ereignisse oder Erfahrungen möglicherweise eine entscheidendere Rolle bei der Erstellung einer Patientenverfügung spielen als stabile individuelle Merkmale.

## Introduction

The United Nations Convention on the Rights of Persons with Disabilities (UN-CRPD) is a human rights treaty that grants persons with disabilities, such as people with dementia (PwD), the freedom to make their own choices (Article 3(1)) [17]. State authorities are obliged to support their ability to make legally valid decisions. This encompasses the right to make decisions regarding a particular medical treatment and to give voluntary, prior and informed consent.

As dementia progresses, PwD can lose their capacity to provide informed consent to complex medical treatment. Despite such loss of capacity, the existence of an available, interpretable and valid advance directive, which is an instrument to express the will of the affected person, can be considered to preserve their autonomy [5, 16]; however, both the decision to create an advance directive and the decisions within an advance directive are complex and cognitively demanding.

In retrospect, the capacity of PwD to provide informed consent at the time of drafting an advance directive is often questioned, thereby casting doubt on the validity of these directives, as the capacity to consent is a prerequisite for the validity of an advance directive. Following a diagnosis of dementia it is often presumed that it is too late to draft an advance directive as even in the early stages of dementia the ability to make complex decisions is frequently compromised. A study found that only one fifth of persons with early stage dementia, most with above-average premorbid intelligence, were deemed competent to complete an advance directive [4].

Advance directives remain insufficiently widespread in many parts of the world. In Germany, only about 51% of people over 60 years old have an advance directive [6]. Another study suggested that 44% of advance directives examined had issues with interpretability and validity [2]. Among PwD, the proportion of unclear cases may be higher but specific research on this issue is currently lacking. Understanding the support needs of individuals with suspected dementia and identifying factors influencing the presence and validity of advance directives are crucial for developing interventions that promote the timely creation of advance directives, ensuring that individuals’ autonomy is upheld even when they can no longer make decisions themselves. Our study addresses the following research questions:

1. What are the proportions of patients with an advance directive, or an advance directive compliant with the informed consent standard, within the population of memory clinic patients?
2. Do patient characteristics, such as age, gender, education, health literacy, need for autonomy, dementia status and comorbidities, predict the existence of advance directives?

This study is a part of the comprehensive research project DECIDE, that aims to enable PwD to exercise their right to self-determination [1].

## Methods

### Setting and participants

We recruited an all-comers sample from the outpatient memory clinics of the University Hospital Frankfurt, Germany and the Klinikum Siegen, Germany, between November 2021 and February 2023.

Patients were eligible for participation in the study if they presented with suspected cognitive impairments. Exclusion criteria were uncompensated severe sensory deficits, insufficient knowledge of the German language or otherwise lack of capacity to consent to research with simultaneous incapacity for supported decision making by a proxy or lack of the participant’s assent.

### Procedure

In both memory clinics participants with suspected dementia usually undergo three appointments: a medical history interview with the treating physician, a neuropsychological assessment and an appointment for communicating the diagnosis. At the medical history interview, we collected information on the outcomes of interest after informed consent to participate in the study had been given. At the time of data collection, the current diagnosis (dementia or other) had not yet been formally established. The description of the sample in relation to dementia severity is based solely on the MMSE (Mini-Mental State Examination) score (Table 1).

**Table 1:** Sample characteristics

Characteristic	*N* = 289
**Age (years)**	72 (64, 79)
**Gender**	
Female	155 (54%)
Male	134 (46%)
**Education**	
School drop out	8 (2.8%)
Volks‑/Hauptschule*	123 (43%)
Mittlere Reife*	70 (24%)
Abitur*	88 (30%)
**Health literacy: “How often do you need help […] reading medical information […]?”**	
Always	36 (13%)
Often	20 (7.0%)
Sometimes	27 (9.4%)
Rarely	51 (18%)
Never	152 (53%)
*(Unknown)*	*3*
**Need for autonomy: “Who should […] make decisions about medical treatment?”**	
Physician alone	4 (1.4%)
Physician considering patient’s opinion	56 (20%)
Physician and patient jointly	162 (57%)
Patient considering physician’s opinion	62 (22%)
Patient alone	2 (0.7%)
*(Unknown)*	*3*
**Somatic comorbidities: CIRS‑G somatic score (SMI)**	4.0 (2.0, 6.0)
*(Unknown)*	*17*
**Minimum CIRS‑G somatic score**	4.0 (2.0, 6.0)
**Psychiatric comorbidities: CIRS‑G psychiatric score**	
0 - no psychiatric disorder	195 (68%)
1 - mild symptoms; no functional impairment	22 (7.6%)
2 - moderate symptoms; mild impact on daily functioning	61 (21%)
3 - severe psychiatric symptoms; significant impairment in function	10 (3.5%)
4 - extremely severe; chronic and disabling psychiatric illness; unable to function independently	0 (0.0%)
*(Unknown)*	*1*
**Cognitive status: MMSE score**	26.0 (22.0, 28.0)
*(Unknown)*	*16*
**Depressive symptoms: GDS score**	3.0 (1.0, 5.0)
*(Unknown)*	*67*

*n* (%); median (IQR)

*German school-leaving qualifications: *Volks‑/Hauptschule* basic/vocational level, *Mittlere Reife* intermediate level, *Abitur* top/academic level

*CIRS‑G* Cumulative Illness Rating Scale - Geriatric, *SMI* somatic morbidity index, *MMSE* Mini-Mental State Examination, *GDS* Geriatric Depression Scale, *IQR* Interquartile Range

### Study design and instruments

We conducted a cross-sectional observational study to investigate the prevalence of advance directives, both with and without compliance with the informed consent standard. While compliance with the informed consent standard is not a legal requirement for creating an advance directive, it is a recommendation of professional societies [3]. Compliance with the informed consent standard means that a capable patient received adequate information to make an informed decision and did so voluntarily [3Chapter 3.2.6, table 3.1, page 40]. To approximate verification of these criteria for existing advance directives, we asked whether, for example, a notary, physician, or psychotherapist had been involved (information), whether capacity to consent had been documented (capacity), and whether the patient had created the directive voluntarily (voluntariness).

To identify potential predictors of advance directive presence, we collected data on age, gender, education, health literacy, need for autonomy in medical decision-making, cognitive status and comorbidities. Health literacy and autonomy were assessed using single items [8, 13]. Somatic and psychiatric comorbidities were evaluated using the German version of the Cumulative Illness Rating Scale-Geriatric (CIRS-G) [7], which includes 13 subscales for somatic conditions and 1 for psychiatric impairments. The dementia diagnosis (if not previously known) was not included in the CIRS‑G score as it had not yet been established at the time of data collection.

Cognitive status was assessed using the Mini-Mental State Examination (MMSE) [10]. Depressive symptoms were measured with the German 15-item short form of the Geriatric Depression Scale (GDS) [11].

### Ethical statements

All procedures followed were in accordance with the Helsinki Declaration of 1975 (in its most recently amended version). Informed consent was obtained from all patients included in the study. The study procedure and material have been reviewed and approved by the Ethics Committee of the Medical Council Westfalen-Lippe (trial no. 2021-518-f-S) and the Ethics Committee of the Medical Faculty at the Goethe University Frankfurt (trial no. 2021-559). The study was registered with the German Registry for Clinical Trials (DRKS00026691).

### Sample size, data analysis and handling of missing data

This study is part of a larger trial, so the sample size was not calculated specifically for the outcomes of interest. With a target of 250 patients, the study achieves a 95% confidence level and a 6.2% margin of error in a worst case scenario.

The first research question involved computing absolute and relative frequencies of advance directives and informed consent compliance criteria, both overall and stratified by patient characteristics. For the second research question (association between advance directives and patient-related characteristics), multivariable logistic regression models were fitted considering compliance with the informed consent standard and without. Potential predictors were demographic variables, need for autonomy in making decisions, health literacy, comorbidity and the severity of dementia as assessed by the MMSE. Differences between sites were assessed using Wilcoxon-Mann-Whitney tests. Likelihood ratio tests assessed categorical predictors with more than two levels.

The 13 somatic subscales of the CIRS‑G [7] were summed to create the somatic morbidity index (SMI), which was treated as a continuous variable. The MMSE score was treated as a continuous variable, while the GDS score was excluded from the statistical analysis due to its correlation with overall psychiatric comorbidity (for details on the instruments see [1]).

In 16 patients the MMSE score was missing, frequently not at random; however, there was no hint of an association between its absence and the presence of an advance directive. Therefore, a significant effect of the MMSE score on the outcomes of interest was excluded in a preliminary complete case analysis (as far as possible) and the main analysis then performed without the MMSE score. Missing SMI items (17 patients) were addressed using a known minimum SMI variable. Sensitivity analyses used multivariate imputation by chained equations (MICE), imputing item-level values and employing the completed SMI.

Statistical analyses used R version 4.3.1 (R Foundation for Statistical Computing, Vienna, Austria), with the MICE package (version 3.16.0, developed by Stef van Buuren, Netherlands) for sensitivity analysis [15]. All tests were two-sided with a 5% significance level, and confidence intervals were reported at the 95% level.

## Results

Among the 306 patients we asked to participate, 293 agreed and were checked for eligibility. Of the patients three had to be excluded due to severe dementia and one due to insufficient knowledge of the German language. Thus, we recruited a total of 289 participants, 152 from Siegen and 137 from Frankfurt.

The median age was 72 years and 54% of the participants were female. The median MMSE score was 26 points. Table 1 shows the basic characteristics of the study sample. There were some significant differences between the patient populations at the two study sites: patients from Frankfurt had higher levels of education and health literacy and suffered less from somatic but more from psychiatric comorbidities (see Supplementary Table S1).

**Fig. 1:**
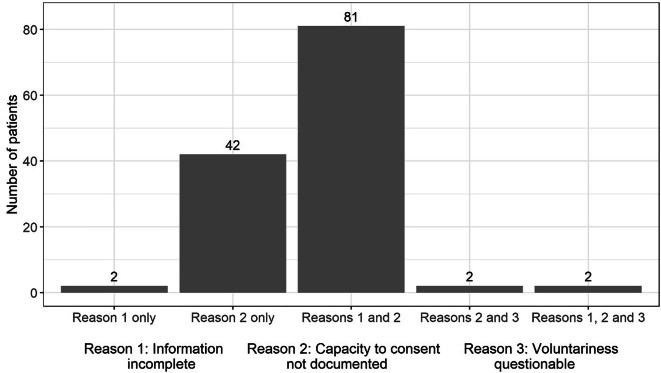
Deficits in meeting the informed consent standard

Regarding our first research question, a total of 170 patients (59%) had an advance directive. Of these, only 41 (24%) complied with the informed consent standard. Figure 1 shows the deficits found in this respect.

Taken together, this means that only 14% of all participants had an advance directive drawn up according to the informed consent standard.

**Fig. 2:**
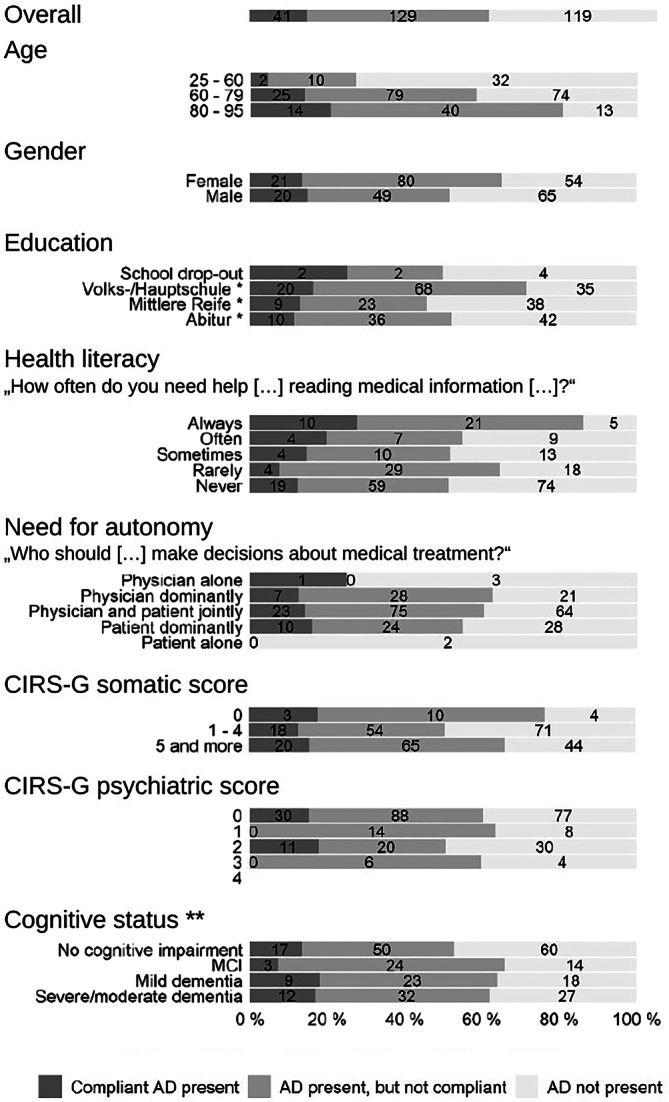
Frequency of advance directives (AD) with consideration of compliance with the informed consent standard * German school-leaving qualifications: Volks-/Hauptschule – basic/vocational level, Mittlere Reife – intermediate level, Abitur – top/academic level ** We classified MMSE scores of 27-30 points as “No cognitive impairment”, of 25-26 points as “MCI (Mild Cognitive Impairment)”, of 21-24 points as “Mild dementia”, and scores below 21 points as “Severe/moderate dementia”

The observed dependence of the frequency of advance directives on patient characteristics is displayed in Fig. 2.

For the second research question, preliminary analyses assessed the relevance of the MMSE score. First, we included the absence of a MMSE score as a covariate in the multivariable model, finding no significant association with advance directive presence (*p* > 0.98). Complete case analyses with the MMSE score as a covariate also showed no significant association (*p* = 0.36 and *p* = 0.15 for main effects; *p* = 0.62 and *p* = 0.96 for site interactions, respectively). Consequently, the MMSE score was excluded as a predictor in further analyses. The results of the main analyses are presented in Tables 2 and 3.

**Table 2:** Results of the multivariable logistic regression model for prevalence of advance directives without consideration of compliance with the informed consent (IC) standard

Predictor	Main effects		Interaction effects with site	
	Odds ratio [95% CI]**	*p*-value	Odds ratio [95% CI]**	*p*-value
*Site *(reference: Siegen)				
Frankfurt	73.54 [0.41, 13,243.78]	0.176	–	–
*Age*				
per increase by 1 year	*1.08 [1.05, 1.12]*	*<* *0.001*	0.97 [0.91, 1.04]	0.412
*Gender *(reference: female)				
Male	0.55 [0.29, 1.04]	0.067	0.46 [0.13, 1.64]	0.231
Education* (reference: Volks‑/Hauptschule)				
School drop-out	0.70 [0.06, 7.81]	0.773	7.41 [0.06, 920.07]	0.416
Mittlere Reife	*0.44 [0.20, 0.98]*	*0.044*	0.55 [0.11, 2.75]	0.465
Abitur	0.59 [0.27, 1.28]	0.181	0.69 [0.15, 3.20]	0.634
*Health literacy: “How often do you need help […] reading medical information […]?” *(reference: never)				
Often or always	0.84 [0.22, 3.18]	0.799	*0.05 [0.00, 0.78]*	*0.032*
Sometimes	0.91 [0.32, 2.62]	0.868	1.07 [0.13, 8.75]	0.950
Rarely	1.52 [0.68, 3.39]	0.309	0.86 [0.17, 4.30]	0.855
*Need for autonomy: “Who should […] make decisions about medical treatment?” *(reference: physician and patient jointly)				
Physician alone or dominantly	0.75 [0.34, 1.64]	0.474	0.86 [0.18, 4.09]	0.849
Patient alone or dominantly	0.89 [0.43, 1.84]	0.750	0.54 [0.13, 2.31]	0.406
*Minimum CIRS‑G somatic score*				
Per increase by 1 score point	1.04 [0.94, 1.16]	0.425	0.90 [0.73, 1.11]	0.311
*CIRS‑G psychiatric score *(reference: 0)				
1 or 2	*2.53 [1.11, 5.77]*	*0.027*	*0.05 [0.01, 0.27]*	*<* *0.001*
3 or 4	3.01 [0.59, 15.32]	0.184	1.07 [0.04, 27.73]	0.967

*German school-leaving qualifications: *Volks‑/Hauptschule* basic/vocational level, *Mittlere Reife* intermediate level, *Abitur* top/academic level

**Multiply adjusted odds ratios relative to the reference level

Italicized values indicate statistically significant results (*p* < 0.05)

*CIRS‑G* Cumulative Illness Rating Scale - Geriatric, *CI* confidence interval

**Table 3:** Results from the multivariable logistic regression model for prevalence of advance directives with consideration of compliance with the IC standard

Predictor	Main effects		Interaction effects with site	
	Odds ratio [95% CI]**	*p*-value	Odds ratio [95% CI]**	*p*-value
*Site *(reference: Siegen)				
Frankfurt	31.93 [0.02, 55,064.50]	0.362	–	–
*Age*				
Per increase by 1 year	*1.06 [1.00, 1.11]*	*0.040*	0.97 [0.87, 1.07]	0.517
*Gender *(reference: female)				
Male	1.52 [0.64, 3.64]	0.347	0.77 [0.13, 4.43]	0.773
Education* (reference: Volks‑/Hauptschule)				
School drop-out	3.59 [0.42, 30.72]	0.243	5.54 [0.08, 405.01]	0.434
Mittlere Reife	1.01 [0.34, 2.97]	0.988	0.64 [0.07, 5.60]	0.690
Abitur	0.73 [0.25, 2.10]	0.560	0.76 [0.09, 6.29]	0.797
*Health literacy: “How often do you need help […] reading medical information […]?” *(reference: never)				
Often or always	0.00 [0.00, ∞]	0.995	0.00 [0.00, ∞]	0.994
Sometimes	0.77 [0.19, 3.15]	0.718	0.92 [0.06, 15.28]	0.954
Rarely	0.00 [0.00, ∞]	0.989	0.00 [0.00, ∞]	0.989
*Need for autonomy: “Who should […] make decisions about medical treatment?” *(reference: physician and patient jointly)				
Physician alone or dominantly	0.75 [0.25, 2.22]	0.600	0.28 [0.03, 2.45]	0.249
Patient alone or dominantly	1.10 [0.39, 3.07]	0.861	0.30 [0.04, 2.39]	0.258
*Minimum CIRS‑G somatic score*				
Per increase by 1 score point	0.89 [0.76, 1.06]	0.188	0.86 [0.61, 1.20]	0.368
*CIRS‑G psychiatric score *(reference: 0)				
1 or 2	1.47 [0.58, 3.70]	0.415	0.41 [0.06, 2.60]	0.344
3 or 4	0.00 [0.00, ∞]	0.994	8.26 [0.00, ∞]	1.000

*German school-leaving qualifications: Volks‑/Hauptschule basic/vocational level, Mittlere Reife intermediate level, Abitur top/academic level

**Multiply adjusted odds ratios vs. reference level

The advance directive prevalence model, excluding informed consent compliance, was based on *N* = 281 records. Age showed the strongest positive main effect, with a nonsignificant interaction with study site. Psychiatric comorbidity (CIRS‑G psychiatric scores 1 and 2 vs. 0) also had a significant positive main effect but a stronger interaction effect reversed the effect in Frankfurt to negative. The contrast for CIRS‑G psychiatric score 3 vs. 0 was nonsignificant, although the variable as a whole was significant (see Table S2). Significant effects were also observed for individual education levels and the interaction between site and health literacy.

Including informed consent compliance enabled one additional record (*N* = 282), with age remaining the only significant predictor.

The sensitivity analyses using multiple imputation confirmed the results of the main analyses.

## Discussion

This study found that 59% of memory clinic patients had advance directives but only 14% complied with the informed consent standard. Age was the strongest predictor of having an advance directive, while psychiatric comorbidities, education and health literacy also played roles; however, these effects varied by site. For informed consent standard compliance, age remained the sole significant predictor. Notably, MMSE scores were unrelated to the presence of advance directives.

The 59% advance directive prevalence in memory clinics aligns with the 51% reported for those aged 60 years and older [6] but exceeds the 29% observed in intensive care unit patients [2]. This discrepancy may stem from our study’s focus on memory clinics, which typically serve an older population. Both our study and those cited [2, 6] found age to be a key factor in having an advance directive. This may reflect older individuals’ greater experience with illness and death but it is not linked to somatic comorbidities, as no association with the SMI was found. Societal norms might also play a role, as older individuals are more likely to be offered the opportunity to create an advance directive.

The study was conducted at two diverse sites to increase participant variety and improve external validity. Siegen’s memory clinic is in a district hospital in a semi-rural area, while Frankfurt’s clinic is part of the University Medical Center in a metropolitan area. The populations at the two sites proved to be different, as shown by varying patient characteristics and the fact that interaction effects often matched the magnitude of main effects in regression analysis. Consequently, nonsignificant overall effects became significant at one site, or significant effects lost significance in site-stratified analyses (see Table S3).

For example, patients from Siegen who would always or often need help reading medical information were significantly more likely to have an advance directive than patients who never did. Conversely, patients from Frankfurt who had graduated from higher secondary schools partly had, dependent on the school type, significantly fewer advance directives than patients with the lower reference level of education. An explanation of both might be that patients with low education and health literacy level are less confident in making ad hoc medical decisions, thus feeling more comfortable when they have an advance directive, created with support by a person considered trustworthy and more competent than themselves. This effect, along with the fact that adherence to the informed consent standard (including counselling) is not a requirement for creating an advance directive under German law, could also explain the different proportions of advance directives compliant with the informed consent standard in Siegen and Frankfurt: as education and health literacy were higher in Frankfurt, participants there might not have seen the necessity to seek any counselling before drafting an advance directive. Another example is the positive association between mild and moderate psychiatric comorbidities and advance directives, observed only in Siegen. This might reflect a diagnostic bias due to disparities in psychiatric care between urban and rural areas. In rural regions, individuals who receive psychiatric diagnoses despite limited access to mental health services may be more proactive and informed about their health, leading to a different patient profile compared to their urban counterparts [9].

None of the patient characteristics we collected, except age, were universal predictors of the existence of advance directives, despite a reasonable sample size. This suggests that incidents or experiences rather than personal traits, may drive people to create an advance directive. Future research could explore what specifically triggers the decision to write an advance directive.

In our study 76% of advance directives did not comply with the informed consent standard, compared to 44% found to be poorly interpretable in another study [2]. It can be assumed that a substantial portion of noncompliant advance directives are still valid under German law. A limitation of our study is the inability to distinguish between valid advance directives and those meeting the informed consent standard. Nevertheless, these findings suggest potential issues with interpretability and adherence, as noncompliant advance directives may face challenges in being properly understood or applied by healthcare providers when needed in critical situations.

## Practical conclusion

Apart from age, specific experiences rather than personal characteristics, may prompt individuals to create an advance directive.

Memory clinics, where dementia is often first diagnosed in its early stages, provide a key opportunity for patients to establish an advance directive while still capable of making autonomous decisions.

This study may serve as a foundation for future research on empowering people with dementia to exercise their right to self-determination in advance directives, particularly in memory clinics.

## Supplementary Information

Supplementary Information includes additional tables presenting subgroup comparisons and statistical test results:Table S1 compares the study populations from Siegen and Frankfurt.Tables S2 and S3 report the results of likelihood ratio tests for categorical variables with more than two levels, applied to models predicting the presence of advance directives and of compliant advance directives, respectively.Table S4 contrasts the significance of main effects in the overall model (presence of ADs, regardless of ICS compliance) with the site-stratified models.

## Data Availability

The anonymized dataset is available upon reasonable request from the corresponding author.

## References

[1] Baisch S et al (2022) Project DECIDE, part 1: increasing the amount of valid advance directives in people with alzheimer’s disease by offering advance care planning—a prospective double-arm intervention study. BMC Med Ethics 23:132. 10.1186/s12910-022-00854-0 36494718 10.1186/s12910-022-00854-0 PMC9733090

[2] De Heer G, Saugel B, Sensen B et al (2017) advance directives and powers of attorney in intensive care patients. Dtsch Ärztebl Int 114:363. 10.3238/arztebl.2017.0363 28625275 10.3238/arztebl.2017.0363 PMC5478788

[3] Deutsche Gesellschaft für Gerontologie und Geriatrie, Deutsche Gesellschaft für Psychiatrie und Psychotherapie, Psychosomatik und Nervenheilkunde (DGPPN), Deutsche Gesellschaft für Neurologie (DGN) (2020) Einwilligung von menschen mit Demenz in medizinische Maßnahmen: Interdisziplinäre S2k-Leitlinie für die medizinische praxis. Kohlhammer, Stuttgart

[4] Fazel S, Hope T, Jacoby R (1999) Dementia, intelligence, and the competence to complete advance directives. Lancet 354(9172):48–10406372. 10.1016/S0140-6736(99)01911-X 10406372 10.1016/S0140-6736(99)01911-X

[5] Goossens B, Sevenants A, Declercq A, Van Audenhove C (2020) Improving shared decision-making in advance care planning: implementation of a cluster randomized staff intervention in dementia care. Patient Educ Couns 103(4):839–847. 10.1016/j.pec.2019.11.024 31818522 10.1016/j.pec.2019.11.024

[6] IfD Allensbach (2014) Haben Sie eine Patientenverfügung verfasst, oder haben Sie vor, das zu tun? (have you written an advance directive, or do you intend to do so?) Statista GmbH. https://de.statista.com/statistik/daten/studie/375267/umfrage/patientenverfuegungen-verbreitung-in-deutschland-nach-altersgruppen/. Accessed 30 Apr 2024

[7] Hock G, Nosper M (2003) Manual CIRS‑G cumulative illness rating scale: Skala zur kumulierten Bewertung von Erkrankungen. Medizinischer Dienst der Krankenversicherung Rheinland-Pfalz, Alzey

[8] Morris NS, MacLean CD, Chew LD, Littenberg B (2006) The single item literacy screener: evaluation of a brief instrument to identify limited reading ability. BMC Fam Pract 7:21. 10.1186/1471-2296-7-21 16563164 10.1186/1471-2296-7-21 PMC1435902

[9] Rommel A, Bretschneider J, Kroll LE, Prütz F, Thom J (2017) The utilization of psychiatric and psychotherapeutic services in Germany—individual determinants and regional differences. J Health Monit 2:3–22. 10.17886/RKI-GBE-2017-122.2 37168125 10.17886/RKI-GBE-2017-122.2 PMC10165906

[10] Schmid NS, Ehrensperger MM, Berres M, Beck IR, Monsch AU (2014) The extension of the German CERAD Neuropsychological assessment battery with tests assessing subcortical, executive and frontal functions improves accuracy in dementia diagnosis. Dement Geriatr Cogn Disord Extra 4:322–334. 10.1159/000357774 10.1159/000357774 PMC4176468 25298776

[11] Sheikh JI, Yesavage JA (1986) Geriatric Depression Scale (GDS): recent evidence and development of a shorter version. Clin Gerontol 5:165–173. 10.1300/J018v05n01_09

[12] Silveira MJ, Kim SYH, Langa KM (2010) Advance directives and outcomes of surrogate decision making before death. N Engl J Med 362:1211–1218. 10.1056/NEJMsa0907901 20357283 10.1056/NEJMsa0907901 PMC2880881

[13] Strull WM, Lo B, Charles G (1984) Do patients want to participate in medical decision making? JAMA 252:2990–2994. 10.1001/jama.1984.03350210038026 6502860

[14] Teno JM, Gruneir A, Schwartz Z et al (2017) Association between advance directives and quality of end-of-life care: a national study. J Am Geriatr Soc 65:1497–1504. 10.1111/jgs.14888 17302654 10.1111/j.1532-5415.2007.01045.x

[15] Van Buuren S, Groothuis-Oudshoorn K (2011) MICE: multivariate imputation by chained equations in R. J Stat Softw 45:1–67. 10.18637/jss.v045.i03

[16] Vollmann J (2001) Advance directives in patients with Alzheimer’s disease; ethical and clinical considerations. Med Health Care Philos 4:161–167. 10.1023/A:1011491100267 11547502 10.1023/a:1011491100267

[17] Wied TS, Knebel M, Tesky VA, Haberstroh J (2019) The Human Right to Make One’s Own Choices – Implications for Supported Decision-Making in Persons With Dementia. Eur Psychol 24(2):146–158

