# Supplementary material for: Advance directives in German memory clinics
Source: Z Gerontol Geriatr. 2025 Jun 27;58(8):681–7. doi: 10.1007/s00391-025-02455-z (PMC12644161; doi:10.1007/s00391-025-02455-z)
Supplement: Supplementary file 1 — Supplementary Information includes additional tables presenting subgroup comparisons and statistical test results:Table S1 compares the study populations from Siegen and Frankfurt.Tables S2 and S3 report the results of likelihood ratio tests for categorical variables with more than two levels, applied to models predicting the presence of advance directives and of compliant advance directives, respectively.Table S4 contrasts the significance of main effects in the overall model (presence of ADs, regardless of ICS compliance) with the site-stratified models. [file 391_2025_2455_MOESM1_ESM.docx]

**Supplementary Material**

**Table S1.** Comparison of the populations from Siegen and Frankfurt.

| Characteristic | Siegen  (N = 152) | Frankfurt  (N = 137) | p-value |
| --- | --- | --- | --- |
| Age | 72 (64, 80) | 72 (64, 79) | 0.5 |
| Gender   Female  Male | 87 (57%) 65 (43%) | 68 (50%) 69 (50%) | 0.2 |
| Education *  School drop-out  Volks-/Hauptschule  Mittlere Reife  Abitur | 5 (3.3%) 84 (55%) 32 (21%) 31 (20%) | 3 (2.2%) 39 (28%) 38 (28%) 57 (42%) | **< 0.001** |
| Health literacy: “How often do you need help […] reading medical information […]?”  Always  Often  Sometimes  Rarely  Never  *(Unknown)* | 36 (24%) 15 (9.9%) 17 (11%) 25 (16%) 59 (39%) — | 0 (0.0%) 5 (3.7%) 10 (7.5%) 26 (19%) 93 (69%) *3* | **< 0.001** |
| Need for autonomy: “Who should […] make decisions about medical treatment?”  Physician alone  Physician considering patient’s opinion  Physician and patient jointly  Patient considering physician’s opinion  Patient alone  *(Unknown)* | 4 (2.7%) 23 (15%) 91 (61%) 29 (19%) 2 (1.3%) *3* | 0 (0.0%) 33 (24%) 71 (52%) 33 (24%) 0 (0.0%) *0* | 0.8 |
| CIRS-G somatic score (SMI)  *(Unknown)* | 4.0 (2.5, 7.0) *1* | 3.0 (2.0, 6.0) *16* | **0.014** |
| Minimum CIRS-G somatic score | 4.0 (2.8, 7.0) | 4.0 (2.0, 6.0) |  |
| CIRS-G psychiatric score  0  1  2  3  4  *(Unknown)* | 119 (78%) 8 (5.3%) 19 (13%) 6 (3.9%) 0 (0.0%) *0* | 76 (56%) 14 (10%) 42 (31%) 4 (2.9%) 0 (0.0%) *1* | **< 0.001** |
| MMSE score  *(Unknown)* | 26.5 (22.0, 29.0) *10* | 26.0 (22.0, 28.0) *6* | 0.3 |
| GDS score  *(Unknown)* | 3.0 (1.0, 5.0) *27* | 3.0 (1.0, 5.0) *40* | 0.8 |

n (%); Median (IQR); Wilcoxon-Mann-Whitney p-value

* German school-leaving qualifications: Volks-/Hauptschule – basic/vocational level, Mittlere Reife – intermediate level, Abitur – top/academic level

**Table S2.** Results of the likelihood ratio tests for significance of the categorical variables having more than two levels in the model for presence of advance directives.

| Variable | df | p-value (χ^2^) of  main effect | p-value (χ^2^) of  interaction |
| --- | --- | --- | --- |
| Education | 3 | 0.227 (4.34) | 0.682 (1.50) |
| Health literacy | 3 | 0.709 (1.39) | 0.123 (5.78) |
| Need for autonomy | 2 | 0.771 (0.52) | 0.704 (0.70) |
| CIRS-G psychiatric score | 2 | **0.044 (6.25)** | **< 0.001 (14.87)** |

**Table S3.** Comparison of significance of (main) effects in the overall model of presence of ADs without consideration of compliance to the ICS to the models stratified by site.

| Predictor | Overall | Siegen | Frankfurt |
| --- | --- | --- | --- |
| **Phase** (Reference: Phase 1) Phase 2 | ***** | ***** | — |
| **Age**  per increase by 1 year | ******* | ******* | ****** |
| **Gender** (Reference: Female) Male | **·** | — | ***** |
| **Education *** (Reference: Volks-/Hauptschule) School drop-out Mittlere Reife Abitur | — ***** — | — — — | — ***** — |
| **Health literacy:** “How often do you need help […] reading medical information […]?” (Reference: Never)  Often or always  Sometimes  Rarely | — — — | ***** — — | — — — |
| **Need for autonomy:** “Who should […] make decisions about medical treatment?” (Reference: Physician and patient jointly)  Physician alone or dominantly  Patient alone or dominantly | — — | — — | — — |
| **Minimum CIRS-G somatic score**  per increase by 1 score point | — | — | — |
| **CIRS-G psychiatric score** (Reference: 0) 1 or 2 3 or 4 | ***** — | ******* — | — — |

Levels of significance: ******* p < 0.001, ****** 0.001 ≤ p < 0.01, ***** 0.001 ≤ p < 0.05, **·** 0.05 ≤ p < 0.1

* German school-leaving qualifications: Volks-/Hauptschule – basic/vocational level, Mittlere Reife – intermediate level, Abitur – top/academic level
